# Intensive Blood Pressure Lowering in Patients With Renal Impairment and Lacunar Stroke

**DOI:** 10.1161/JAHA.119.013637

**Published:** 2019-08-19

**Authors:** Stephen Makin, William N. Whiteley

**Affiliations:** ^1^ Centre for Rural Health University of Aberdeen Glasgow United Kingdom; ^2^ Centre for Clinical Brain Sciences University of Edinburgh United Kingdom

**Keywords:** Editorials, blood pressure, renal disease, stroke prevention, Ischemic Stroke

## Abstract

See Article Agarwal et al

There is a complex relationship between higher blood pressure, chronic kidney disease (CKD), and the risk of stroke. Higher blood pressure is associated with a higher risk of incident ischemic stroke and more strongly with a higher risk of incident hemorrhagic stroke.^1^ Although patients with CKD are at a higher risk of stroke, there is not a consistent monotonic relationship between higher blood pressure and higher risk of cardiovascular diseases in patients with CKD; indeed, low or normal blood pressures have been associated with a higher risk of incident cardiovascular diseases in some studies. This relationship may be causal, but also could be explained by reverse causality.^2^

Patients with CKD seem to be at particularly high risk of stroke. There is an inverse linear relationship between glomerular filtration rate and the risk of stroke, with an ≈7% increased relative risk of stroke for every 10–mL/min per 1.73 m^2^ decrease in glomerular filtration rate, which seems consistent across major stroke subtypes.^3^

In addition, it has been hypothesized that strokes caused by cerebral small‐vessel disease (“lacunar strokes”) are part of a multisystem small‐vessel disorder that affects both the cerebral and the renal circulations. At younger ages, there are associations between renal impairment and cerebral white matter hyperintensities and other markers of cerebral small‐vessel disease, although whether this is a manifestation of risk factors common to stroke and CKD (chiefly hypertension) or other mechanisms is uncertain.^4^, ^5^, ^6^

For these reasons, the effect of intensive blood pressure reduction on stroke incidence in patients with CKD is of particular interest for clinicians and epidemiologists.

In this issue of the *Journal of the American Heart Association* (*JAHA*), Agarwal and colleagues^7^ present a secondary analysis of data from the SPS3 (Secondary Prevention of Small Subcortical Strokes) trial, reporting the effect of intensive blood pressure reduction in patients with recent lacunar stroke with and without CKD (defining CKD as estimated glomerular filtration rate <60 mL/min per 1.73 m^2^).^8^ The SPS3 trial was a factorial trial that randomly allocated patients with a recent magnetic resonance imaging–defined lacunar stroke to a systolic blood pressure target of <130 mm Hg versus 130 to 149 mm Hg; and to aspirin versus aspirin and clopidogrel. An important blood pressure difference was achieved between groups at 1 year (ie, an 11–mm Hg difference in systolic blood pressure). With more intense blood pressure lowering, a modest but nonsignificant reduction in incidence of all stroke, the primary outcome, was observed (hazard ratio, 0.84; 95% CI, 0.64–1.03).

In this secondary report of the SPS3 trial, patients with CKD had a higher risk of recurrent stroke, as expected. However, there was no qualitative or statistical evidence of a difference in the formally neutral effect of intensive blood pressure lowering on death, stroke, myocardial infarction, or intracranial hemorrhage between patients with CKD and patients without CKD.

This is consistent with the results of the SPRINT (Systolic Blood Pressure Intervention Trial), which did not demonstrate a modification in the beneficial effect of intensive versus less intensive blood pressure lowering on cardiovascular outcomes in patients with and without CKD.^9^ In SPRINT, intensive blood pressure lowering was also not associated with a lower quality of life. Prior neutral trials of intensive blood pressure lowering in patients with CKD may have included too few participants to detect any positive effects on myocardial infarction or stroke.^10^, ^11^

Therefore, the present study does not support different blood pressure targets for patients with lacunar stroke with and without CKD. This is of relevance for physicians looking after patients with recent stroke. For physicians looking after patients with CKD, further trials of intense BP lowering targeting this group have been suggested to dispel remaining clinical uncertainties about the balance between potential harms of blood pressure lowering, particularly to the kidney, and the potential benefits of blood pressure lowering, reducing incidence of myocardial infarction, stroke, and progression of renal disease.^2^

## Disclosures

None.
